# Bibliographical

**Published:** 1884-12

**Authors:** 


### Bibliographical.

### The Student's Manual of Histology, for the use of Students, Practitioners, and Microscopists. Third Edition.

Entirely Re-written; Greatly Enlarged, and Newly Illustrated. By Charles H. Stowell, M.D.F.R.M.S., Professor of Histology and Microscopy, and in charge of the Histological Laboratory of the University of Michigan. Illustrated 178 New Engravings.

This volume is just from the press, published by the author. It is a work claiming the special attention of those for whom it has been prepared. The appearance of this, the third edition, so soon after the second, is evidence of the popularity and value of the work. It is a hand-book, invaluable to the microscopist and the student. Professor Stowell's ability for the preparation of such a work is not excelled, if equaled by any one in this country. His attention has been very largely, if not almost exclusively, occupied with this branch of science for many years past, not only in its study and investigation, but he has been actively engaged in teaching for a number of years. He has also been in direct communication with the microscopists of this country, and it has been, and is his pride to make every available thing, tributary to his work. In the preface, he says: "Histologists and microscopists have not been idle during the past two years, and, as a result, many important facts have been added to our knowledge of the structure of tissues." No one, perhaps, in the country has made a closer study of this particular branch of the subject than Dr. Stowell. In regard to this edition, he says: "The text has been entirely re-written, and a large amount of new matter added. Many of the poorer illustrations have been replaced by new cuts, and new figures illustrate important points. Five chapters have been added to the text, treating on subjects previously omitted, or lightly passed over. The methods of examining, given at the close of each chapter, have been largely increased, although, as announced in the first preface, only those methods are given that are most familiar, and that have proved the most satisfactory in my hands." In this edition of the work not only are the tissues of the various structures presented and fully described, but the method of preparing and examining such specimens, is very fully presented. These present the most advanced methods of manipulation and treatment. No one can study this work, and follow its direction in investigation and manipulation, without attaining a good degree of familiarity with this rapidly developing subject, and I have no hesitation in recommending it as the best manual for the student of this department of science, with which I am acquainted. It should be in the hands of every member of the dental profession who makes any pretension to the knowledge of its science. The work may be obtained through any book seller, or by addressing Professor C. H. Stowell, Ann Arbor, Mich. Price, $3.50.

